# Genetic and environmental contributions to individual differences in visual attention and oculomotor control in early infancy

**DOI:** 10.1111/cdev.14185

**Published:** 2024-10-24

**Authors:** Monica Siqueiros‐Sanchez, Giorgia Bussu, Ana Maria Portugal, Angelica Ronald, Terje Falck‐Ytter

**Affiliations:** ^1^ Department of Women's and Children's Health, Center of Neurodevelopmental Disorders (KIND), Centre for Psychiatry Research Karolinska Institutet & Stockholm Health Care Services, Region Stockholm Stockholm Sweden; ^2^ Department of Psychiatry and Behavioral Sciences Stanford University School of Medicine Stanford California USA; ^3^ Development and Neurodiversity Lab, Department of Psychology Uppsala University Uppsala Sweden; ^4^ School of Psychology University of Surrey Guildford UK

## Abstract

Infants differ in their level of eye movement control, which at the extreme could be linked to autism. We assessed eye movements in 450 twins (225 pairs, 57% monozygotic, 46% female, aged 5–6 months) using the gap‐overlap eye‐tracking task. Shorter latency in the gap condition was associated with having more parent‐rated autistic traits at 2 years. Latency across the task's three conditions was primarily explained by one highly heritable latent factor likely representing individual differences in basic oculomotor efficiency and/or in visual information processing. Additionally, disengagement of attention was linked to unique genetic factors, suggesting that genetic factors involved in visual attention are different from those involved in basic visual information processing and oculomotor efficiency.

**Abbreviations**
ADHD: attention deficit and hyperactivity disorder
AIC: Akaike's information criteria
ASD: autism spectrum disorder
BATSS: Babytwins Study Sweden
BIC: Bayes information criteria
CS: central stimuli
DZ: dizygotic
GEE: general estimating equation
GPS: genome‐wide polygenic score
GWAS: genome wide association study
ICC: intra‐class correlation
MZ: monozygotic
PS: peripheral stimuli
Q‐CHAT: Quantitative Checklist for Autism in Toddlers
SES: socioeconomic status
SNP: single‐nucleotide‐polymorphism

Our perception and experience of our environment is influenced by the manner in which we allocate our attention. Eye movements serve as a visual attentional filter, influencing which information will be further processed and which will be disregarded (Amso & Scerif, 2015). Prior to language acquisition and during the early stages of motor development, eye movements are informative of infants' attention allocation as they are readily available means of exploration (Colombo et al., 1991). The ability to voluntarily shift gaze from an engaging stimulus to another emerges as early as 3 months of age and is known as visual disengagement (or disengagement, for short) (Johnson et al., 1991). Successfully executing a gaze shift and visually disengaging from stimuli can influence later cognitive processes and developmental skills, including social skills (Veer et al., 2017).

To understand the genetic underpinnings of behaviors like visual attention, researchers often use twin studies. By comparing the similarity in traits between identical and non‐identical twins, these studies can estimate the proportion of variance in a trait due to genetic factors relative to environmental factors (Rijsdijk & Sham, 2002). This proportion is known as heritability. Twin studies also allow researchers to test how much of the covariation between two traits is explained by genetic factors and the extent to which these genetic influences are shared between traits.

Studies have shown that individual differences in eye movements and attention have moderate to high heritability. In a recent meta‐analysis of infant twin studies, attention functions showed a significant pooled twin heritability of 48% (95% CI, 0.15–0.75; *p* = .003) (Austerberry et al., 2022). Twin and family studies indicate moderate heritability of individual differences in eye movements (*h* ^2^ = 0.38–0.55, 95% CIs [0.21–0.76]) (Siqueiros Sanchez, Falck‐Ytter, et al., 2020; Siqueiros Sanchez, Pettersson, et al., 2020; Vaidyanathan et al., 2014) and attention (*h* ^2^ = 0.30–0.80) (Brikell et al., 2015; Siqueiros Sanchez, Falck‐Ytter, et al., 2020). Limited research suggests that different types of gaze and eye movement behaviors in infancy are heritable. Two studies found substantial heritability of eye and mouth preference in 5‐month‐old infants and 18‐ to 24‐month‐old toddlers (Constantino et al., 2017; Viktorsson et al., 2023), whereas two other studies found low heritability for face orienting (Portugal et al., 2024) and moderate heritability for face preference (Portugal et al., 2024) and for a right gaze bias when viewing faces (Viktorsson et al., 2024). However, these studies examined visual attention to social stimuli, not gaze‐shifts in non‐social contexts. Nonetheless, the evidence supports the potential of eye movements, and derived metrics, as cognitive and clinical endophenotypes.

Furthermore, research suggests that a subset of genetic influences underlying variability in cognitive functions and in clinical traits may be shared. For instance, shared genetic factors have been observed between response inhibition measures (including the go/no‐go task and the antisaccade eye tracking paradigm) and attention deficit and hyperactivity disorder (ADHD) traits (Falck‐Ytter et al., 2020; Kuntsi et al., 2014; Siqueiros Sanchez, Falck‐Ytter, et al., 2020).

In the context of developmental research, longitudinal twin designs are particularly valuable. They allow for estimating the sources of individual differences of early attention and testing any associations between said sources to later‐emerging traits, such as autism symptoms in toddlerhood. Additionally, recent advances in molecular genetics, specifically the development of genome‐wide polygenic scores (GPSs), provide a means to assess an individual's genetic susceptibility to traits, disorders, or diseases (Ronald, 2020) and for testing potential associations between behaviors (e.g., eye movements) and genetic liability to a disorder (GPS), prior to the emergence of the disorder's observable symptomatology. Although there are not currently well‐powered GPS for infant behavioral traits, GPS for neurodevelopmental conditions are of interest in relation to early attention (Ronald & Gui, 2024).

## Eye movements as potential endophenotypes

Specific oculomotor behaviors and associated attentional functions have been considered as potential endophenotypes for psychiatric disorders and neurodevelopmental conditions. An endophenotype is a heritable trait thought to provide a causal link between a phenotype and its genetic underpinnings (Gottesman & Gould, 2003).

Current research shows differences in looking behavior between typically developing controls and individuals with neurodevelopmental conditions, including ADHD (Munoz et al., 2003; Siqueiros Sanchez, Falck‐Ytter, et al., 2020) and autism spectrum disorder (ASD) (Elison et al., 2013; Elsabbagh et al., 2013), as well as psychiatric disorders including schizophrenia (Hutton & Ettinger, 2006; Munoz & Everling, 2004; Wolf et al., 2021).

Independent lines of research show preliminary evidence of associations between biological and behavioral measures (including neural connectivity or microstructure, motor development and pupillometry) and genetic susceptibility to psychiatric conditions such as ADHD, ASD, and schizophrenia (Hermosillo et al., 2020; Portugal et al., 2022; Serdarevic et al., 2020). Evidence of shared genetic factors between eye movements and psychiatric traits, as well as associations between behavioral measures and psychiatric genetic liability, further strengthens the potential of eye movements as endophenotypes for psychiatric conditions.

## Visual attention and ASD

Atypicalities in visual attention could reflect general differences in brain development in autism, which are widespread (Hazlett et al., 2017) and extend far beyond social brain networks (Elsabbagh & Johnson, 2016). It is even possible that differences in visual attention play an active role in the developmental pathways linked to autism. Specifically, it has been suggested that atypicalities in attention disengagement in infancy could lead to differences in visual perception, arousal regulation, and social interaction, which, over time, could contribute to the development of autistic symptoms (Elsabbagh et al., 2013; Falck‐Ytter & Bussu, 2023; Keehn et al., 2013).

In support of *atypical* disengagement as a predictor of ASD, a series of studies in early childhood show a link between longer gaze shift latencies (slower disengagement) and ASD liability or diagnosis (Bryson et al., 2017; Elison et al., 2013; Elsabbagh et al., 2013; Landry & Bryson, 2004). Importantly, atypicalities in visual disengagement seem to already be present in the first year of life, preceding the onset of autism symptoms. However, the findings on the link between disengagement and ASD in later developmental stages are mixed (Johnson et al., 2016; Sacrey et al., 2014; Siqueiros Sanchez, Pettersson, et al., 2020). These discrepancies have been attributed to differences in stimuli (i.e., static vs. dynamic, novel vs. non‐novel) and/or age (Sacrey et al., 2014) since the development of visual attention follows a non‐linear trajectory. At group level, gaze shifting stabilizes at around 3–6 months (14 weeks) of age and visual disengagement at around 4‐to‐6 months (18–22 weeks) of age (Butcher et al., 2000; Hood & Atkinson, 1993; Hunnius et al., 2008). At the individual level, the stability of gaze shifts is highly stable after ~4 months (18 weeks), whereas the stability of visual disengagement is still low at 6 months (26 weeks) (Butcher et al., 2000).

One hypothesis posits that, given the rapid brain development undergone from infancy to late‐childhood, compensatory mechanisms or strategies may obscure atypicalities in visual orienting and disengagement in older individuals with ASD (Elsabbagh & Johnson, 2007). Despite its putative association to ASD in early infancy, the extent to which disengagement during the first year of life is a heritable trait has never been investigated.

## The gap‐overlap paradigm

Gaze shifts and visual disengagement have been widely studied using the Gap‐Overlap eye tracking paradigm (Johnson et al., 2016; Jones et al., 2014). This paradigm includes three different experimental conditions: gap, baseline, and overlap. Each condition measures, in units of time, the ease with which the participant disengages its gaze from a visual stimulus and orients it to a new one. The conditions putatively capture distinct attention‐related processes (Fischer & Breitmeyer, 1987), as they elicit gaze shifts of different durations and are associated with different neural correlates (Csibra et al., 1997). For example, target‐ and saccade‐locked event‐related potentials measured by EEG showed a larger P1 effect in gap trials relative to in the overlap trials, suggesting enhanced activation of the extrastriate visual cortex during non‐engaged attention; similarly, a parietal positivity was associated with the offset of the target in the gap trials, but with the initiation of the saccade in overlap trials (Csibra et al., 1997).

Furthermore, individual differences in gaze shifts elicited by the Gap‐Overlap task may be partly underpinned by different genetic factors. In a previous study on school‐aged children, multivariate twin models showed that a significant portion of genetic influences on individual differences in gaze shift latencies elicited by each condition are shared across conditions, whereas others are unique to each (Siqueiros Sanchez, Pettersson, et al., 2020). However, whether this is also the case in infancy or whether there is an association between gaze shifts in infancy and genetic susceptibility to ASD is not known.

Therefore, while substantial progress has been made in understanding the heritability of eye movements and their potential role as endophenotypes for neurodevelopmental conditions, key questions remain. This study aims to investigate the heritability of visual disengagement in infancy and its potential link to genetic susceptibility for ASDs, leveraging both twin study methodologies and advances in polygenic risk scoring in a 5‐month‐old cohort of infant twins from the Babytwins Study Sweden (BATSS) (Falck‐Ytter et al., 2021).

The aim of the study was twofold: (1) To assess the relative influence of genetic and environmental factors on individual differences in gaze shift latencies, including visual disengagement, during the Gap‐Overlap task and (2) to examine the association between gaze shift latencies and ASD. Specifically, we tested whether gaze shift latencies and disengagement at 5 months were associated with ASD traits at 24 and 36 months. In addition, we investigated the link between early attention and the genetic likelihood of ASD as measured by GPSs.

To test for disorder‐specific associations between eye metrics and genetic likelihood of ASD, rather than to psychopathology in general, we included GPS for ADHD and schizophrenia, two disorders with known visual attention atypicalities (Kuntsi et al., 2014; Siqueiros Sanchez, Falck‐Ytter, et al., 2020; Wolf et al., 2021) and (2) the high degree of shared genetic factors across these psychiatric disorders (Pettersson et al., 2016). Beyond neurodevelopmental and psychiatric phenotypes, we also included GPS scores for educational attainment and IQ, due to the observed links to attentional measures (Polderman et al., 2006), and for physical height as a control.

Our specific hypotheses were that individual differences in all eye tracking measures would be explained by a combination of moderate genetic influences, moderate‐to‐high unique environmental influences, and low but significant shared‐environmental influences. We hypothesized genetic influences based on our previous findings using the gap‐overlap paradigm in older children (Siqueiros Sanchez, Pettersson, et al., 2020) and recent studies showing substantial heritability for various early‐life gaze behaviors (albeit with a different paradigm) (Constantino et al., 2017). Still, we also expected shared environmental factors to play a role, as twin studies generally indicate their greater influence in early life, often explaining patterns in twin correlations for cognitive functions (Plomin et al., 2016; Polderman et al., 2015), even in infancy (Austerberry et al., 2022). We also hypothesized positive associations between later ASD traits and (1) gaze shift latencies in the overlap condition and (2) with disengagement scores (Elison et al., 2013; Elsabbagh et al., 2013). We expected slower disengagement to be linked to polygenic likelihood of ASD. We considered this analysis explorative considering the likely low statistical power for testing this correlation in the sample (Bogdan et al., 2018). The power of polygenic analyses is tied to, among other factors, the size and age of the genome wide association study (GWAS) it was generated from (Choi et al., 2020), which in case of autism is, at the time of publication, limited (Martin et al., 2019).

## METHOD

### Sample

Recruited participants belonged to the Babytwins study, a study featuring a longitudinal cohort of infant twins (Falck‐Ytter et al., 2021). Infants included in this cohort were recruited from 2016 to 2020 from the greater Stockholm metropolitan area and identified through their national population registry. The majority of the infants in the Babytwins cohort had at least one parent who was born in Sweden (89.7%), were born to parents holding a university degree, and came from a moderate‐to‐high socioeconomic status (SES) household. Due to Swedish societal norms, parental birthplace was collected in lieu of race or ethnicity. Further details on the recruitment strategy and full sample demographics are reported in the cohort's protocol paper (Falck‐Ytter et al., 2021). The inclusion criteria of the cohort required twins to be 5–6 months old at the time of the visit, to have been reared together, and to have at least one parent with detailed knowledge of the pregnancy (thus excluding surrogacy but not in‐vitro) and medical history, and with fluency in Swedish. Exclusion criteria were opposite‐sex twin pairs, known diagnosis of epilepsy, genetic syndrome, or medical conditions affecting brain development or compromising the child's ability to participate in the study, vision or hearing impairments, and premature birth (<34 weeks). Additional inclusion criteria for this study was valid eye tracking data for both twins.

Of the total cohort sample (311 pairs), 225 pairs (128 monozygotic [MZ; 45.31% female]; 97 dizygotic [DZ; 48.45% female]) were included in this study, reflecting exclusion and inclusion criteria. Twenty‐eight twins were excluded due to parent‐reported twin‐to‐twin transfusion syndrome (12 pairs), report of seizures at the time of birth (2 individuals from different pairs), very low birth weight (<1.5 kg, 1 individual), and report of spina bifida (1 individual). Twenty‐three infants did not take part in the eye‐tracking assessment due to technical reasons (3 pairs), lack of time (1 pair plus 1 individual), bad calibration (1 individual), and tiredness (4 pairs plus 5 individuals). Seventy‐four infants (37 pairs) were excluded since they had less than five trials in at least one condition of the Gap‐Overlap task. Summary and dispersion metrics for the total sample in this study are presented in Table 1. In person, data collection was performed at the Centre of Neurodevelopmental Disorders at Karolinska Institutet (KIND) in Stockholm, Sweden. Written informed consent was obtained from the infants' legal guardians. This study was approved by the regional ethics board in Stockholm and was conducted in accordance with the Declaration of Helsinki.

**TABLE 1:** Study sample characteristics.

Variable	Mean (SD)	Range min, max	Skewness	Kurtosis
Demographics				
Sex (F = 210 pairs [46.7%])	‐	‐	‐	‐
Age	167.54 (8.86) days [5.58 months]	145 days [4.83 months], 203 days [6.76 months]	0.59	0.58
Eye tracking measures				
Gaze shift latencies (ms)				
Gap	309.69 (45.23)	216.57, 512.40	1	1.66
Baseline	360.83 (64.04)	245.43, 643.89	1.16	2.09
Overlap	511.76 (116.14)	296.20, 908.14	0.50	−0.23
Difference scores (ms)				
Facilitation [Baseline minus Gap]	51.14 (45.22)	−90.40, 282.11	0.80	3
Disengagement [Overlap minus Baseline]	150.94 (95.80)	−54.44, 493.31	0.58	−0.02

Abbreviations: F, female; max, maximum; min, minimum; ms, milliseconds; SD, standard deviation.

### Procedure

Eye‐tracking data analyzed in this study was collected using the Gap Overlap task (described in the following section) at the 5‐month time point of the longitudinal BATSS study, along with several other paradigms and assessments not related to the current research. Eye movements were recorded using a TX‐300 Tobii eye‐tracker. Stimuli were displayed on a 23″ monitor with a 1920 × 1080 pixel resolution. The participant sat on their caregiver's lap or on a car seat placed on the caregiver's lap facing the eye tracker at approximately 65 cm from the display screen. The height and angle of the eye tracker were adjusted to ensure optimal gaze position. Prior to task display, a 5‐point infant‐friendly manually controlled calibration sequence composed of colorful dynamic shrinking spirals (6°–0.5° diameter) was run. Eye tracking data collection began only after a successful calibration was achieved (according to the experimenter—with calibration repeated if necessary). The full testing battery can be found in the cohorts protocol paper (Falck‐Ytter et al., 2021). The experimenter administering the task remained out of sight but were able to monitor the infant's behavior in real time through a camera placed on top of the eye‐tracker. Experimenters were able to redirect the infant's attention if needed with auditory attention grabbers embedded in the script.

### Gap Overlap task

The Gap‐Overlap task is a visual attention shifting paradigm used to measure visual disengagement. The task includes three conditions: Gap, Baseline and Overlap. Common to all conditions is the central stimulus appearing on the screen followed by a different peripherally appearing stimulus. Conditions differ on the onset and offset of the central stimulus in relation to the peripheral stimulus onset: the central stimulus offsets 200 ms before the onset of the peripheral stimulus creating a stimuli‐free gap in between stimuli (Gap); the central stimulus offsets simultaneously to the onset of the peripheral stimulus (Baseline); and the central stimulus remains displayed throughout the onset and presentation of the peripheral stimulus (Overlap).

The Gap‐Overlap task included here was an updated infant friendly version of the task (Elsabbagh et al., 2013; Johnson et al., 1991). This version featured attractive and dynamic stimuli (a constricting‐expanding cartoon clock) against a pink background and a gaze‐contingent modality for the peripheral stimulus (which was randomly picked between a set of a sun, cloud, ball, star, dog), meaning stimuli remained on display until the infant fixated on them. The central stimuli (CS) ranged between 2.1° × 2.1° and 3.3° × 3.3° visual degrees (°) in width, whereas the peripheral stimulus was 2.5° × 2.5° wide and appearing at a 19° eccentricity of visual angle from the center. The task included a random 600–700 ms Inter‐Stimuli Interval (interval between gaze being identified in the central stimulus and peripheral stimulus onset starting) and the screen side (right/left) where the peripheral stimulus was displayed was randomized.

### Analysis of eye tracking data

All data were processed and analyzed offline using MATLAB (MathWorks) scripts designed for the Eurosibs study (Jones et al., 2019). Raw eye tracking data were segmented around trial on/offset event markers. When available, data from left and right eyes were averaged, otherwise only one eye was used. For a trial to be considered as valid, and thus included in the analysis , the quality of the data needed to be sufficient to compute a gaze shift latency and for the infant to execute the expected gaze shift (from the central, CS, to peripheral stimuli, PS). The following criteria had to be met: (1) a minimum of 24 ms of gaze on the CS and the PS to count as a shift to either stimulus; (2) no more than 200 ms of total gaze samples could be missing when attending the central stimulus; (3) no more than 50 ms periods of consecutive gaze samples could be missing when attending the central stimulus; (4) no gaze shifts toward the opposite direction after exiting the central stimulus; and (5) latency duration was in the 150–1200 ms range. Trials not meeting these criteria were excluded.

Using only valid trials, the variables computed from this task were: mean gaze shift latency in the Gap, Baseline, and Overlap conditions, and the facilitation and disengagement scores. Gaze shift latency was defined as the elapsed time between the onset of the peripheral target and the gaze entering the area of interest surrounding the peripheral target; facilitation was defined as the mean Baseline gaze shift latency minus mean Gap gaze shift latency; and disengagement as the mean Overlap gaze shift latency time minus mean Baseline gaze shift latency (Elsabbagh et al., 2013). Only infants with at least 5 valid trials in each condition were included in the analyses.

### Twin analyses

In the classic twin design, phenotypic similarity within twin pairs is assessed separately for MZ and DZ twins. Thus, the classic twin design involves the comparison of within pair similarity of MZ twins on a trait, who are genetically identical with the within pair similarity of same‐sex DZ twins on a trait, who on average share ~50% of their segregating genes. For example, if MZ twin pairs are more similar to one another than DZ twin pairs, this can be attributed to genetic effects. The sources of variation in a trait can be partitioned into genetic [heritability; additive (A) and/or nonadditive (D)] and environmental influences. Environmental influences can be further partitioned into shared environment (C; environmental influences that make children growing up in the same family similar) and unique (non‐shared) environment (E; environmental influences that make children growing up in the same family different). Values for heritability estimates, as well as those for environmental influences, range from zero to one.

In this study, estimates for the relative contributions of genetic and environmental influences were computed for all eye tracking variables (average gaze shift latency in the Gap, Baseline and Overlap conditions, and facilitation [Baseline minus Gap] and disengagement [Overlap minus Baseline]) scores. First, intra‐class correlations (ICCs) of all main variables were computed separately for MZ and DZ twins. A series of saturated models were fitted to test for twin model assumptions of mean and variance equality across zygosity and twin order. To estimate the heritability of performance on each variable, based on the pattern of ICCs (see Results for more details) a series of ACE univariate biometric twin models were fitted together with nested (AE, CE, and E) models.

Univariate twin model analyses were followed by multivariate twin model analyses to assess independence of the genetic and environmental influences on latencies elicited by the different conditions. In multivariate twin analyses, cross‐twin cross‐trait correlations are used to partition the covariance between traits (in this case latencies from each condition) into genetic and environmental influences. Three types of models were fitted in order of decreasing complexity: the correlated factors model, the independent pathway model, and the common pathway model (Rijsdijk & Sham, 2002). In the correlated factors model, which is the least constrained model, the sources of variance (genetic and environmental) are allowed to correlate between phenotypes, these correlations (*r*) can range from zero (no overlap) to 1 (complete overlap). In the more restricted independent pathway model, genetic and environmental factors influence the response variables separately. In the common pathway model, the most constrained of all the models, it is assumed that a single latent phenotypic factor influences variation among the response variables; this single latent factor is in turn influenced by genetic and environmental factors. Prior to twin modeling, age, sex, and SES (operationalized as maternal level of education) were regressed gaze shift latencies. Gaze shift latencies were then transformed using the square root transformation. To examine model goodness of fit and select the best model, we used Akaike's information criteria (AIC) for univariate models, and Bayes information criteria (BIC) for multivariate models; lower AIC and BIC values indicate a better fit (Markon & Krueger, 2004; Raftery, 1995). All twin modeling analyses were carried out in R using the OpenMx package (version 2.18.1 with NPSOL optimizer, for univariate) (Neale et al., 2016).

### The Quantitative Checklist for Autism in Toddlers

The Quantitative Checklist for Autism in Toddlers (Q‐CHAT) is a parent report, 25‐item screening questionnaire that assesses autistic traits dimensionally in toddlerhood (Allison et al., 2008). The Q‐CHAT is a quantitative revision of the Checklist for Autism in Toddlers (Baron‐Cohen et al., 1992) screening questionnaire for ASD. It uses a Likert scale format to assess each trait in terms of frequency per day. The Q‐CHAT domains encompass social communication, repetitive, stereotyped, and sensory behaviors. Q‐CHAT scores were collected at both 24 and 36 months. The Q‐CHAT was originally designed to screen for ASD in toddlers aged 18–24 months. However, research has demonstrated that the Q‐CHAT can effectively identify autistic traits in children up to 37 months old (Allison et al., 2008). Imputation of missing scores was done to replace missing values (34%) at 36 months (see Supporting Information).

### Genome‐wide polygenic scores

DNA data were collected via saliva samples from all the participating twins as part of the BATSS protocol (Falck‐Ytter et al., 2021). In addition to confirming zygosity, the samples were genotyped with the Illumina Infinium array (Illumina, San Diego, CA, USA) to calculate GPS for neurodevelopmental conditions, schizophrenia, IQ, educational attainment, and height. The GPS included in this study were calculated using polygenic prediction via Bayesian regression and continuous shrinkage priors (Ge et al., 2019) and were based on the following GWAS: ASD (Grove et al., 2019), ADHD (Demontis et al., 2019), schizophrenia (The Schizophrenia Working Group of the Psychiatric Genomics Consortium et al., 2020), educational attainment (Lee et al., 2018), IQ (Savage et al., 2018), and physical height (Yengo et al., 2018). To control for genetic ancestry, as the base rate of specific SNPs can vary across populations and can thus affect validity of the GPS analyses, a principal component analysis (PCA by EIGENSOFT 7.2.1) was performed based on the BATSS full cohort and two reference datasets: the HapMap Phase III (International HapMap Consortium, 2003), representing individuals with genetic ancestry from Asia, Africa and Europe, and SweGen (Ameur et al., 2017), representing the genetic ancestry of the study's country Swedish population. The sample was predominantly of European ancestry, except for one pair. Complete procedures for quality control of the included GPS can be found in the cohort's BATSS protocol paper (Falck‐Ytter et al., 2021).

### Analysis of associations between eye tracking measures, and GPSs and later ASD traits (Q‐CHAT) using general estimating equation models

General estimating equation (GEE) models were used to estimate the associations between our five eye tracking variables with (1) common genetic likelihood of ASD and other phenotypes and (2) with ASD traits at 24 and at 36 months. For the first analyses, GWAS‐based GPS of ASD, ADHD, schizophrenia, educational attainment, IQ and physical height (as a negative control) served as dependent variables and each eye tracking variable served as the independent variables. The first 10 principal components of ancestry were also included as covariates in these models.

For the second analysis, Q‐CHAT total scores at 24 months (*n* = 308) and at 36 months (*n* = 450) served as dependent variables and our five eye tracking variables served as independent variables. Age, sex, and maternal SES were regressed on the five eye tracking variables and on Q‐CHAT scores prior to GEE modeling on both sets of analyses. GEE analyses of 24‐ and 36‐month data were conducted independently.

GEE models allow us to account for the non‐independence of the twin data in this sample by using cluster‐robust SEs with the Sandwich Estimator. GEE analyses were done in R using the drgee package (Zetterqvist & Sjölander, 2015). Multiple comparisons were addressed by applying a Bonferroni correction, adjusting the *α* value (.05) according to the number of tests performed.

## RESULTS

### Genetic and environmental influences on gaze shift latencies and difference scores in 5‐month‐old infants

**FIGURE 1:**
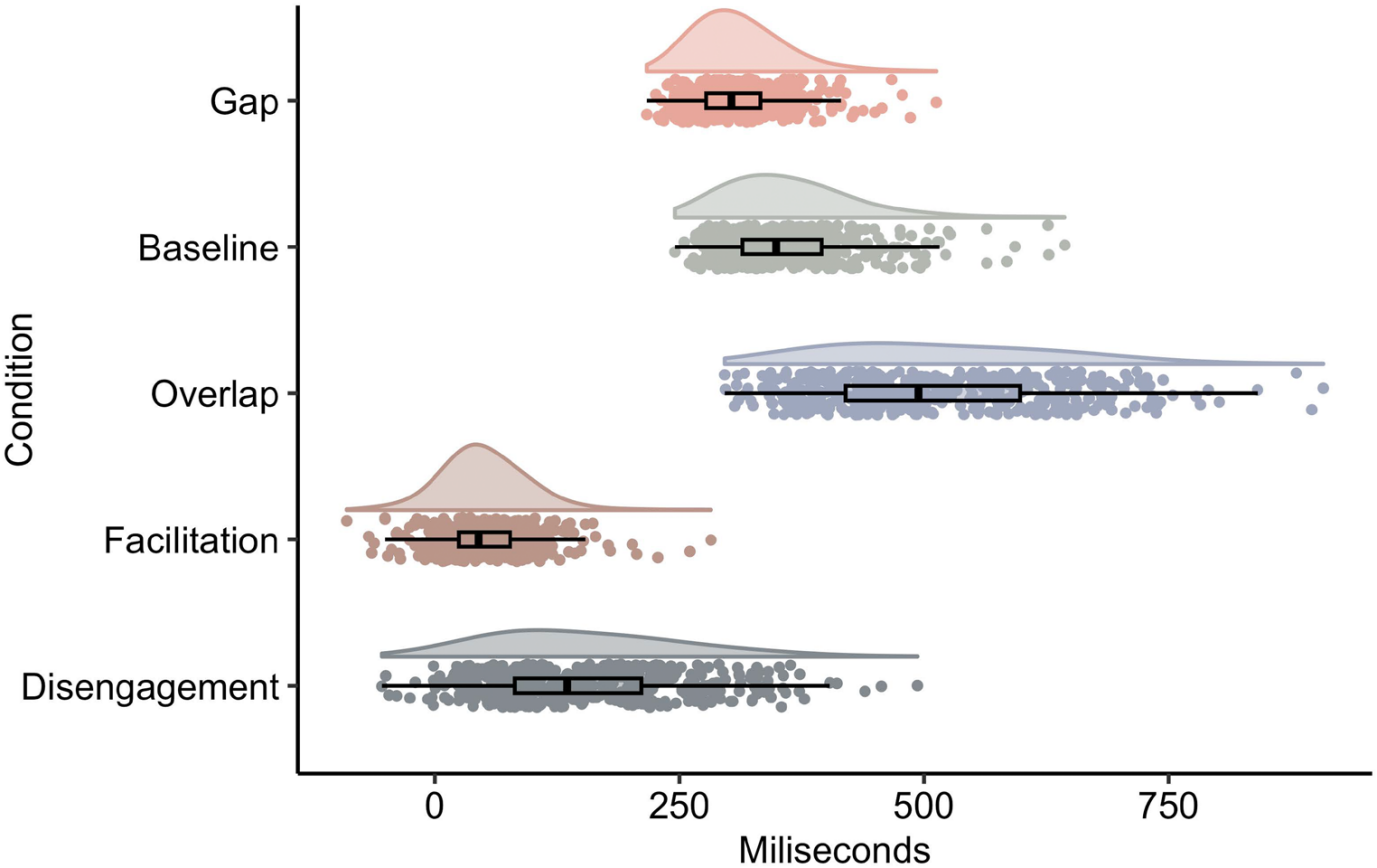
Gaze shift latencies for gap, baseline and overlap conditions, and difference scores of facilitation (Baseline‐Gap) and disengagement (Overlap‐Baseline) in milliseconds (ms).

Gaze shift latencies were within the expected durations for each condition in infancy and normally distributed (Table 1; Figure 1).

#### Univariate twin analyses

**FIGURE 2:**
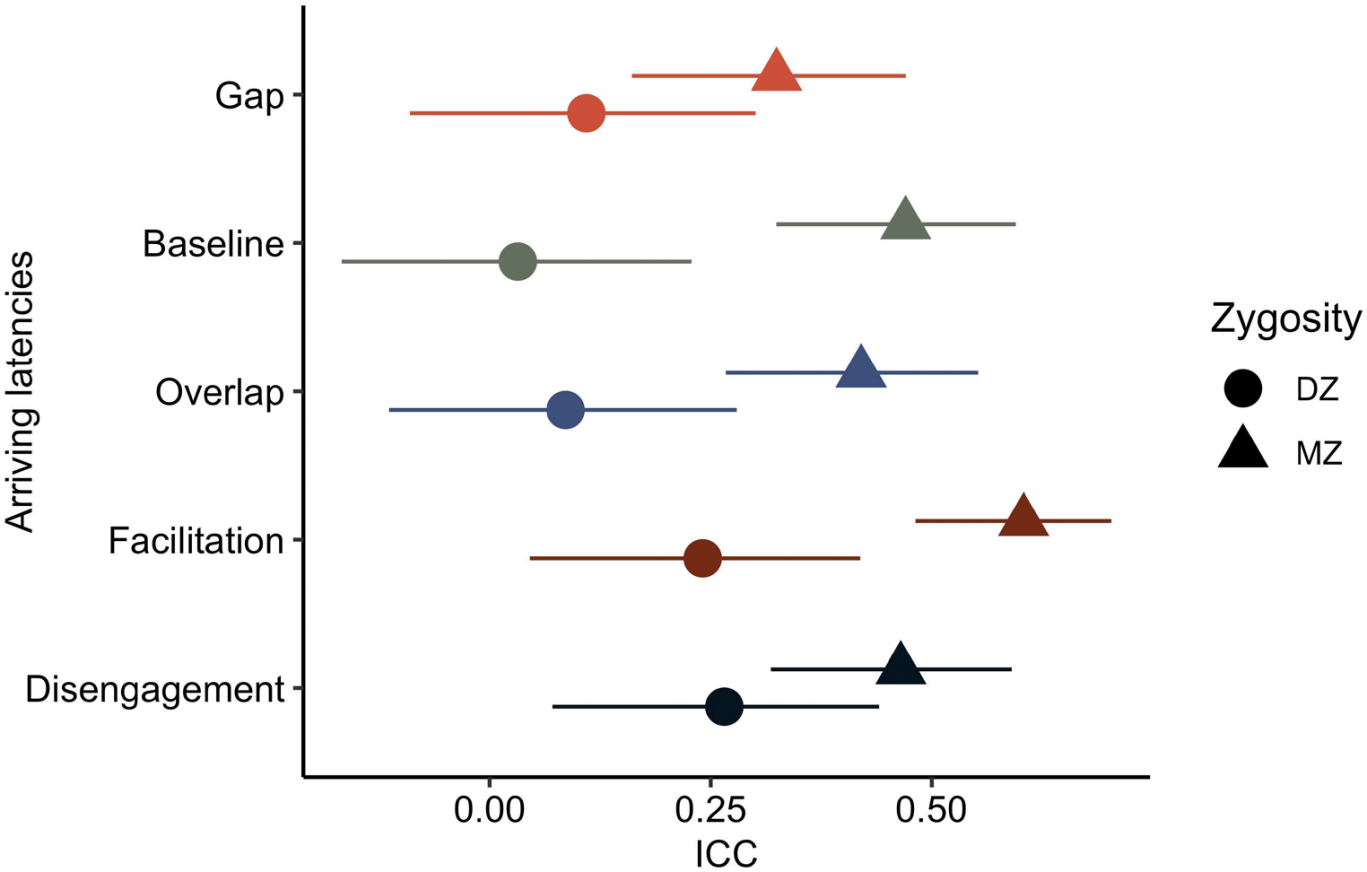
Intraclass correlations of gaze shift latencies from Gap task conditions and scores for MZ and DZ twins. DZ, dizygotic twins; ICC, intra‐class correlations; MZ, monozygotic twins. 95% confidence intervals are represented by horizontal lines crossing circles and triangles.

In line with our hypothesis, the MZ twin ICC coefficients were larger than those for the DZ twins. Because MZ ICCs were roughly twice as large as DZ ICCs across all conditions and mean difference scores (Figure 2), non‐additive genetic factors are not likely to play a substantial role. Therefore, an ACE model (which estimates additive genetic variation in addition to shared and non‐shared environmental factors) was selected for univariate twin modeling analyses rather than an ADE model (which estimates both additive and non‐additive genetic variation and non‐shared environmental factors but not shared environmental factors).

Saturated models testing for homogeneity of means, variance, and covariance between co‐twins and across zygosity groups showed that most homogeneity assumptions were met except for the assumption of equal variance across zygosity groups for gaze shift latencies in the baseline condition (Tables S1–S5). The results from univariate ACE and nested (AE, CE, and E) models (Tables S6–S10) showed that, contrary to our hypothesis, an AE model was identified, based on AIC criteria, as the best fit for all eye tracking variables indicating that shared environmental influences could be dropped from the models (Table 2). Heritability estimates and non‐shared environmental effects were, as hypothesized, moderate (*h* ^*2*^ = 0.40–0.60; *e* ^2^ = 0.40–0.60).

**TABLE 2:** Genetic and environmental contributions to gaze shift latencies and derived scores from best fitting models (AE).

	Model	Genetic	Environment	
		*h* ^*2*^	*c* ^2^	*e* ^2^
Gap	AE	0.50 [0.37, 0.61]	0 (0,0)	0.50 [0.39, 0.63]
Baseline	AE	0.60 [0.48, 0.69]	0 (0,0)	0.40 [0.31, 0.52]
Overlap	AE	0.43 [0.28, 0.56]	0 (0,0)	0.57 [0.44, 0.72]
Facilitation	AE	0.40 [0.25, 0.53]	0 (0,0)	0.60 [0.47, 0.75]
Disengagement	AE	0.44 [0.28, 0.57]	0 (0,0)	0.56 [0.43, 0.72]

#### Multivariate twin analyses

**FIGURE 3:**
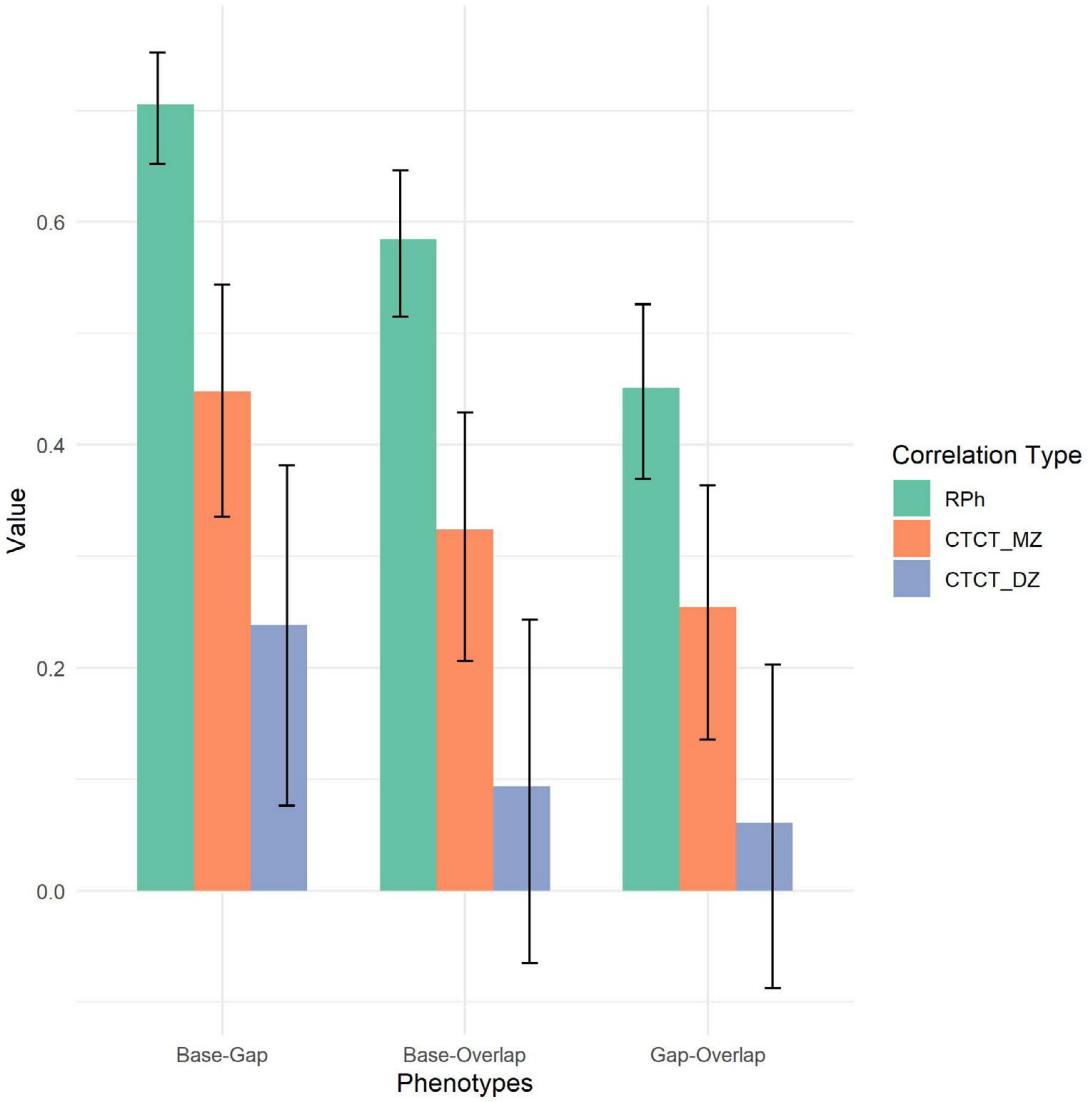
Phenotypic correlations (in green) and cross‐twin cross‐trait correlations (CTCT) split between monozygotic (MZ; in orange) and dizygotic twins (DZ; in purple). Phenotypic correlations refer to within‐individual (i.e. twin 1) between‐trait correlations (i.e. latency in Baseline condition—latency in Gap condition). Cross‐twin cross‐trait correlations give insight into sources of covariances; these represent the strength of the relationship between one trait (i.e. latency in Baseline condition) of twin 1 and another trait (i.e. latency in Gap condition) of twin 2. A larger cross‐twin cross‐trait correlation in MZ (in orange) than in DZ (in purple) twins suggests that the phenotypic correlation between the two traits is partially owing to common genetic influences. An MZ cross‐twin cross‐trait correlation that is less than the phenotypic correlation (in green) suggests a contribution of unique environmental factors to the phenotypic correlation between the two traits. Error bars are showing 95% confidence intervals.

Exploratory multivariate twin analyses were conducted to assess the independence of the genetic and environmental influences on mean gaze shift latencies in each condition of the Gap‐Overlap task. Cross‐twin cross‐trait correlations suggested possible familial influences on the covariance between mean gaze shift latencies to the different task conditions (Figure 3). The common pathway model (see Method) was identified as the best model solution as it provided a better fit than the correlated factors and independent pathway solutions based on BIC criteria without differing significantly from the saturated model (Table 3).

**TABLE 3:** Multivariate model fitting statistics for each multivariate model tested on gaze shift latencies in the three task conditions (i.e., baseline, gap, overlap), compared to the fully saturated model.

Model	n^o^ parameters	−2LL	df	AIC	BIC	ΔLL	Δdf	*p*
Fully saturated	54	3193	1296	3301	3486	‐	‐	‐
Correlated factors	21	3237	1329	3279	3350	43.25	33	0.11
Independent pathway	21	3237	1329	3279	3350	43.25	33	0.11
Common factor	**18**	**3240**	**1332**	**3276**	**3337**	**46.76**	**36**	**0.11**

*Note*: The selected model solution (in bold) was the one with the lowest BIC, also non‐significantly different from the fully saturated model.

Abbreviations: −2LL, log‐likelihood fit statistics; AIC, Akaike information criterion; BIC, Bayesian information criterion; df, degrees of freedom; Δdf, difference in degrees of freedom from the reference model; ΔLL, difference in log‐likelihood fit statistics from the reference model.

**FIGURE 4:**
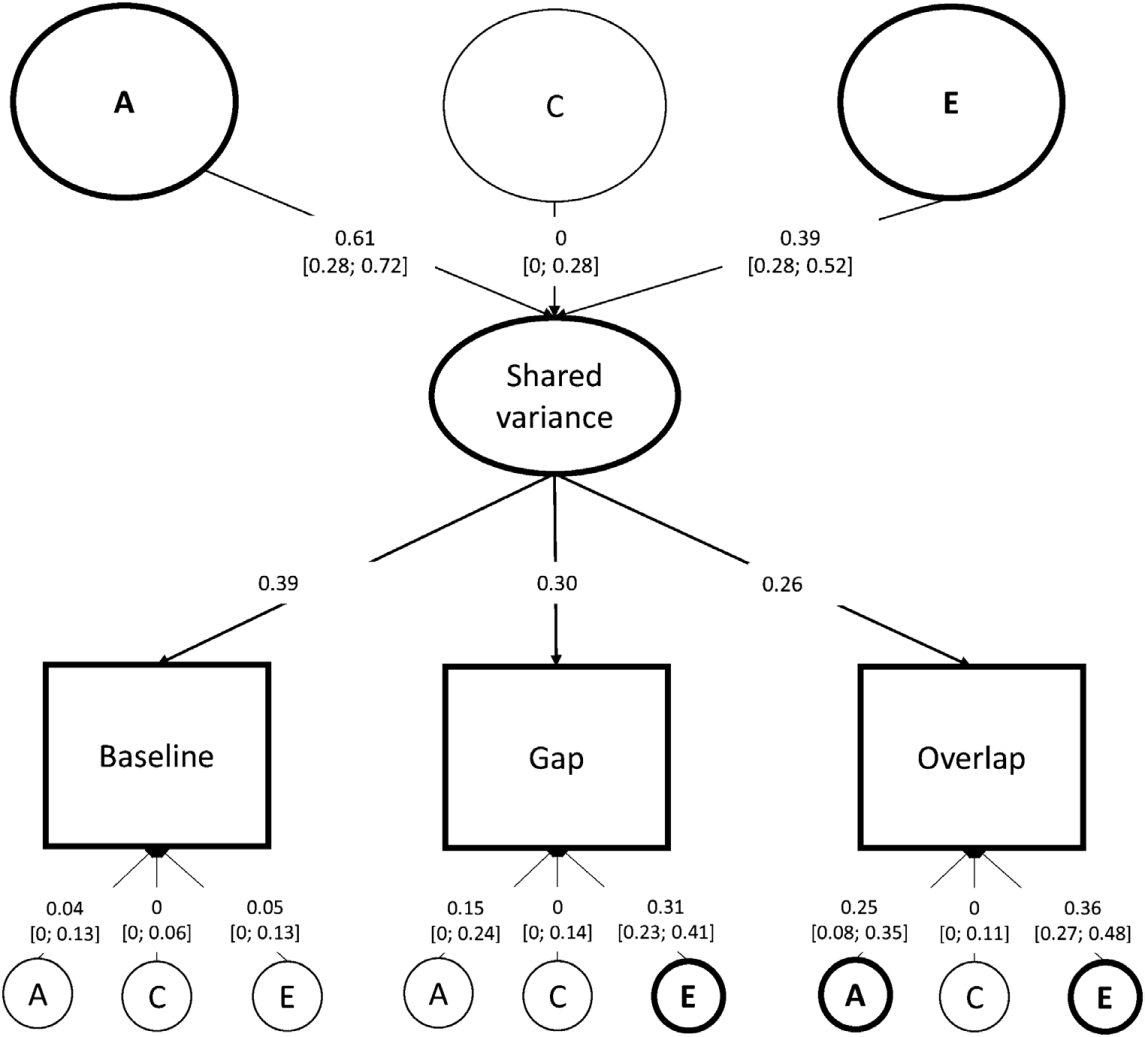
Common pathway model solution to the multivariate model of mean gaze shift latency in each condition of the Gap‐Overlap task. Observed measures are represented by squares, and latent factors by circles. Variance partitions (with 95% confidence intervals) and factor loadings are reported on the edges.

**FIGURE 5:**
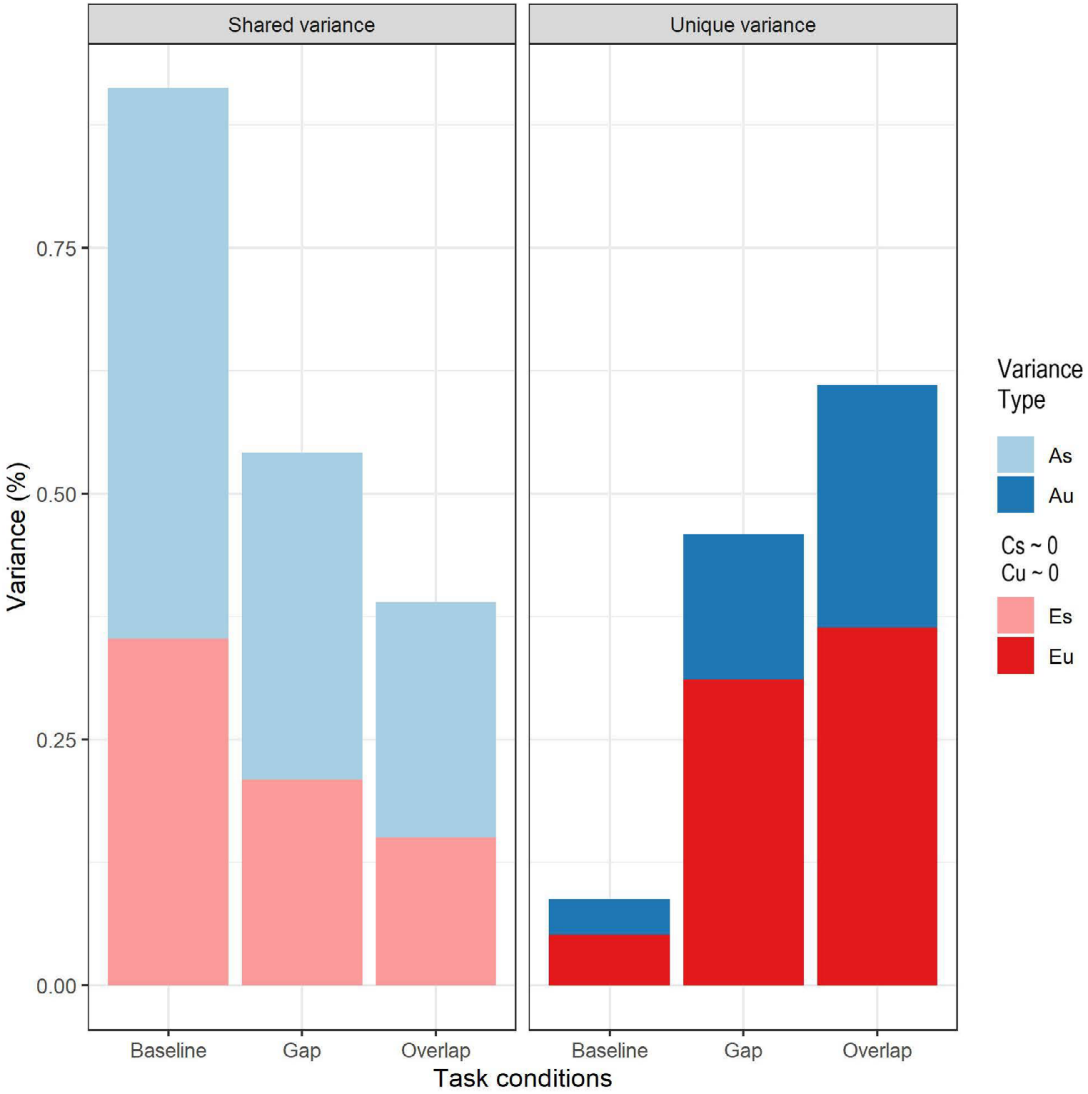
Variance partition into genetic (in blue) and environmental components (in red) divided by variance type, i.e., shared across different task conditions, or unique to each condition. A_s_, % shared variance explained by additive genetics; A_u_, % unique variance explained by additive genetics; C_s_, % shared variance explained by shared environment; C_u_, % unique variance explained by shared environment; E_s_, % shared variance explained by unique environment; E_u_, % unique variance explained by unique environment. Shared environment (C_s_/C_u_) did not explain any significant % of the variance.

In the identified common pathway solution, a single latent factor accounted for the shared variance among mean gaze shift latencies from conditions in the Gap‐Overlap task (see Figure 4 for a schematic representation of the model solution). This latent factor was influenced by additive genetics (*h* ^2^ = 0.613 [0.278; 0.721]) and non‐shared environmental factors (*e* ^2^ = 0.387 [0.279; 0.521]), without significant influences from shared environmental factors (*c* ^2^ = 0 [0; 0.283]). This latent factor explained a substantial amount of the variance in mean gaze shift latencies from each condition (Baseline = 0.91; Gap = 0.54; Overlap = 0.39) (Figure 4 depicts factor loadings rather than estimates of shared variance). Decomposition of the shared variance to mean gaze shift latencies in each condition showed low‐to‐moderate effects of shared genetic factors ($A_{s}^{\text{Baseline}}$ = 0.559 [0.249; 0.681]; $A_{s}^{\mathrm{Gap}}$ = 0.332 [0.150; 0.428]; $A_{s}^{\text{Overlap}}$ = 0.239 [0.107; 0.319]) and low‐to‐moderate effects of shared unique environmental factors ($E_{s}^{\text{Baseline}}$ = 0.353 [0.252; 0.483]; $E_{s}^{\mathrm{Gap}}$ = 0.209 [0.150; 0.289]; $E_{s}^{\text{Overlap}}$ = 0.151 [0.105; 0.212]). Contributions from shared environmental factors were not significant for the latencies elicited by any of the conditions ($C_{s}^{\text{Baseline}}$ = 0 [0; 0.257]; $C_{s}^{\mathrm{Gap}}$ = 0 [0; 0.158]; $C_{s}^{\text{Overlap}}$ = 0 [0; 0.114]). Figure 5 illustrates the decomposition of shared (and non‐shared) variance for mean gaze shift latencies across all conditions.

The common pathway solution also indicated significant contributions of non‐shared factors to the residual variance of some conditions (bottom of Figures 4 and 5). In the baseline condition, there were no significant influences of unique genetic or environmental influences ($A_{u}^{\text{Baseline}}$ = 0.037 [0; 0.129]; $C_{u}^{\text{Baseline}}$ = 0 [0; 0.06]; $E_{u}^{\text{Baseline}}$ = 0.051 [0; 0.128]). In the gap condition, residual variance was only influenced by non‐shared environment ($E_{u}^{\mathrm{Gap}}$ = 0.312 [0.230; 0.410]), whereas unique influences of genetics and shared environment were not significant ($A_{u}^{\mathrm{Gap}}$ = 0.147 [0; 0.238]; $C_{u}^{\mathrm{Gap}}$ = 0 [0; 0.143]). Gaze shift latencies from the overlap condition showed a substantial amount (~60%) of unique residual variance, with contributions from additive genetics ($A_{u}^{\text{Overlap}}$ = 0.246 [0.083; 0.350]), and non‐shared environment ($E_{u}^{\text{Overlap}}$ = 0.364 [0.269; 0.535]); unique influences from shared environment in this condition were not significant ($C_{u}^{\text{Overlap}}$ = 0 [0; 0.114]).

### Associations between gaze shift latencies and difference scores with GPS and ASD traits

A series of GEE analyses tested putative associations between eye tracking measures from the Gap‐Overlap task with ASD traits in toddlerhood (Figure S1) and GPS for neurodevelopmental conditions, schizophrenia, IQ, and educational attainment (with height as a control). Contrary to our hypotheses, we did not find an association between gaze shift latencies in the overlap condition or the disengagement score and GPS for neurodevelopmental (ASD, ADHD) or psychiatric diagnoses (schizophrenia), IQ, and educational attainment (Table S11) or autistics traits at either age (Tables 4 and 5).

**TABLE 4:** Summary statistics of the association analysis between eye‐tracking scores and later autistic traits at 24 months.

Task condition	*β*	SE	*p*‐Value
Baseline	−.14	.06	.01
Gap	−.21	.06	.0001
Overlap	−.101	.06	.1
Facilitation	.011	.06	.87
Disengagement	−.03	.06	.65

Note: The table shows results from the Generalized Estimating Equations testing associations between arriving gaze latency scores for the different eye‐tracking task conditions tested at 5 months of age and the total score from the Quantitative Checklist for Autism at 24 months of age (*n* = 308). Results are reported on separate models in each row as regression coefficient (*β*), standard error, and uncorrected *p*‐value. Scores were residualized by sex, testing age, and maternal socioeconomic status (scaled before being entered into the model). Results are deemed significant for *p* < *α* with Bonferroni corrected *α* = .005 based on the 10 tests performed.

**TABLE 5:** Summary statistics of the association analysis between eye‐tracking scores (infancy) and autistic traits measured at 36 months.

Task condition	*β*	SE	*p*‐Value
Baseline	−.08	.05	.09
Gap	−.12	.05	.012
Overlap	−.06	.05	.26
Facilitation	−.004	.05	.93
Disengagement	−.01	.05	.78

Note: The table shows results from the Generalized Estimating Equations testing associations between arriving gaze latency scores for the different eye‐tracking task conditions tested at 5 months of age and the total score from the Quantitative Checklist for Autism at 36 months of age (*n* = 450; 155 imputed scores). Results are reported on separate models in each row as regression coefficient (*β*), standard error, and uncorrected *p*‐value. Scores were residualized by sex and testing age (scaled before being entered into the model). Results are deemed significant for *p* < *α* with Bonferroni corrected *α* = .005 based on the 10 tests performed.

However, gaze shift latencies from the Gap condition were negatively associated to parent rated ASD traits at 24 months (*β* = −.21, *p* uncorrected = .0001; Table 4; Figure S2). In other words, infants with higher ASD traits at follow up tended to move their gaze quicker to the peripheral target in the gap condition. Similarly, gaze shift latencies in the Baseline and the Gap conditions were marginally negatively associated with ASD traits at 24 months (*β* = −.14, *p* uncorrected = .01; Table 4) and 36 months (*β* = −.12, *p* uncorrected = .012; Table 5), respectively. However, these marginal associations did not survive adjusting for additional comparisons with other eye tracking measures (results are deemed significant for *p* < *α* with Bonferroni corrected *α* ≤ .005 based on the 10 tests performed). We did not find any significant associations between mean gaze shift latencies for any of the other conditions of the Gap‐Overlap task, nor the derived disengagement and facilitation scores with ASD traits at 24 (Table 4) nor at 36 (Table 5) months.

## DISCUSSION

In this study, we examined the relative contribution of genetic and environmental effects to oculomotor measures from the Gap‐Overlap task and their association to autistic traits in toddlerhood and to common genetic variants for neurodevelopmental conditions, schizophrenia and cognitive traits using a classic twin design. As hypothesized, we found evidence for moderate genetic effects (*h* ^2^ = 40%–60%) and unique environmental effects (*e* ^2^ = 40%–50%) on eye movements in the context of non‐social stimuli and low‐level visual attention in early infancy (Table 2; Tables S6–S10). Surprisingly, given that heritability often increases with age, these heritability estimates were similar to those reported in school aged children (*h* ^2^ = 38%–55%) rather than lower (Siqueiros Sanchez, Pettersson, et al., 2020), although they are similar to a pooled twin heritability estimate of infant attention functions from a recent meta‐analysis (39%; Austerberry et al., 2022). Similarly, unique environmental effects were similar to those from older children (*e* ^2^ = 40%–60%) who also performed the Gap‐Overlap task (Siqueiros Sanchez, Pettersson, et al., 2020) (Table 2; Tables S6–S10). While unique environment effects may include measurement error, the relatively high heritability speaks to the reliability of the measures studied here.

Contrary to our hypothesis, we found no evidence of shared environment influences in any of the measures. Although some twin studies on psychometric traits (i.e., IQ) suggest significant contributions from shared environment in early life (Plomin et al., 2016; Polderman et al., 2015), our results are in line with those from previous twin studies of eye movements (Portugal et al., 2022; Siqueiros Sanchez, Falck‐Ytter, et al., 2020; Siqueiros Sanchez, Pettersson, et al., 2020; Viktorsson et al., 2023) and of early cognitive ability in infants (Bussu et al., 2023) reporting none or marginal influence of shared environment.

Multivariate analyses of gaze shift latencies of the Gap‐Overlap task conditions showed that a common, moderately heritable, latent factor explained most of the covariance across these (Table 3; Figures 4 and 5). This is in line with a previous study in 9‐to‐11‐year‐old twins assessed on the Gap‐Overlap task (Siqueiros Sanchez, Pettersson, et al., 2020). This factor could capture individual differences in oculomotor processes common to all conditions which are prerequisites to successfully executing gaze shifts and orienting (Atkinson et al., 1992). Other potential explanations include individual differences in visual information processing speed (Csibra et al., 1997; Jacobson et al., 1992) or efficiency of the visual and vestibular systems subserving the oculomotor system (Dorris et al., 1997, 2002; Munoz & Schall, 2003).

We also found evidence of unique genetic and environmental influences in gaze shift latencies from the overlap condition, suggesting distinct biological and environmental factors underlying disengagement in the presence of competition in infancy. This is in contrast to the aforementioned study with school‐aged twins where the authors did not find unique genetic influences for this condition (Siqueiros Sanchez, Pettersson, et al., 2020). This difference may be attributable to different factors. For example, genetic influences could be more prominent in this condition in infancy compared to late childhood, or to the measure may be more sensitive to individual differences in attentional control (as captured by disengagement) in early infancy, or to differences in how latencies were computed formerly (time elapsed between the onset of the peripheral target and the gaze leaving the area of interest surrounding the central target) versus in the present study. Our current results suggest that in infancy, genetic factors that are linked to attentional control (i.e., disengagement) are partly independent from the genetic factors linked to basic oculomotor control and visual processing speed required for visual orienting in the absence of competing stimuli (as in the gap and baseline conditions). Interestingly, also in the social domain there is evidence suggesting genetic differentiation between different types of social looking in infancy (Falck‐Ytter, 2024).

Contrary to our hypotheses, gaze shift latencies in the overlap condition and the disengagement score were not associated with later ASD traits or ASD GPS. Thus, our study did not indicate that specific genetic effects observed in the overlap condition at 5 months are associated with trait‐level autism in the general population. It may be that atypical disengagement (linked to the overlap condition specifically) emerges later in infancy (7–14 m) among infants with high levels of ASD traits (Elison et al., 2013; Elsabbagh et al., 2013). This could be due to disengagement being linked to different brain areas than those supporting basic attentional and perceptual processes, such as frontal areas linked to executive attention (Johnson et al., 2021).

We did, however, find a negative association between ASD traits at 24 months and gaze shift latencies in the gap condition. This correlation survived correction for multiple testing. There was also a trend level (non‐significant) association between the gap condition and ASD traits at 36 months and between the baseline condition and 24 months ASD traits, although these did not survive correction for multiple testing. Although the only formally statistically significant association was found for the gap condition, the pattern of results does not suggest that the association is specific to this condition. Rather, it seems more likely that the association may be due to shared processes captured, to a different extent, by all conditions (e.g., visual or oculomotor processing speed). Elison et al. (2013) found that latencies in both the gap and the overlap conditions discriminated between infants at elevated likelihood of ASD versus infants at low likelihood of ASD at 7 months of age, again suggesting an effect that was not linked to a specific experimental condition. The same study also found that individual differences in the gap condition in typically developing infants were associated with differences in white matter microstructure in tracts linking the brainstem and the cortex (cortico‐spinal tract). Notably, although we found *shorter* latencies to be linked to higher levels of trait‐level ASD at follow up, Elison et al. found that infants at elevated likelihood of ASD had *longer* latencies, and no specific association to later ASD classification. This difference between study results may be linked to differences in the age of the studies samples, as previous research into early signs of autism in the visual domain has often seen age*group interaction effects on infant phenotypes (Jones & Klin, 2013; Nyström et al., 2018, 2019).

We did not find associations between the eye tracking measures and GPS. Results from association analyses with GPS are to be interpreted cautiously. GWAS capture only a portion of a trait's genetic predisposition, known as single‐nucleotide‐polymorphism (SNP) heritability, which has been identified from recent GWAS. Consequently, GPSs derived from GWAS exclude other forms of genetic variation, likely resulting in lower heritability estimates compared to twin studies (Choi et al., 2020). Additionally, power in GPS analyses is related to a number of factors, including the sample size of the GWAS that the GPS was generated from (Choi et al., 2020) and the underlying strength of association present between the GPS and the phenotype. Autism is a highly genetically heterogeneous condition that at present shows modest SNP heritability in the most recently published GWAS (Grove et al., 2019). Given these caveats, ruling out any potential associations to eye tracking measures from the Gap‐Overlap task is premature as other studies featuring GPS derived from larger GWAS (Hermosillo et al., 2020; Portugal et al., 2022) or a larger target sample (~1000) (Serdarevic et al., 2020) have found small but significant associations.

Two further limitations are the sample size, as it may be underpowered to detect small effects, and the generally high SES of the infants in the BATSS cohort (Falck‐Ytter et al., 2021). Although we controlled for SES during analyses, the high SES limits the generalizability of these results to samples with broader levels of income and educational attainment and may differentially impact eye metrics in this task (Siqueiros Sanchez et al., 2021) but this negative result may reflect characteristics of this sample (being very young and not at elevated likelihood of ASD).

## CONCLUSION

Our results show that individual differences in gaze shift latencies largely reflect genetic differences between the infants, with a substantial fraction of these genetic influences overlapping across latencies from all conditions. This latent factor was moderately heritable (*A* = 0.613, CI [0.278, 0.721]) and likely to reflect individual differences in processes linked to basic oculomotor control and visual processing speed. Gaze shift latencies in the overlap condition specifically were also linked to unique genetic influences (unique *A* = 0.246, CI [0.083, 0.350]). These influences may be linked to the unique attentional component captured by the overlap condition (disengagement cost). We did not find evidence of a link between visual disengagement and common genetic likelihood of ASD nor a phenotypic association with autistic traits, as measured by an ASD screening instrument. However, we did find an association between gaze shift latencies in the Gap condition and, marginally, in the Baseline condition, and ASD traits at 24 months. This finding suggests a link between basic and early‐emerging visual or oculomotor processing and later ASD traits.

## CONFLICT OF INTEREST STATEMENT

The authors have no conflicts of interest to disclose.

## ETHICS STATEMENT

This study was approved by the regional ethics board in Stockholm and was conducted in accordance with the Declaration of Helsinki.

## Supporting information


Data S1.

## Data Availability

The deidentified data that support the findings of this study are available from the corresponding author, T.F.Y., upon reasonable request in compliance with applicable privacy laws, data protection and requirements for consent and anonymization. Most of the analytic code necessary to reproduce the analyses presented in this work is publicly available, any other custom code is available from the corresponding author, T.F.Y., upon reasonable request. The analyses presented here were not preregistered.
