# Supplementary material for: Genetic and environmental contributions to individual differences in visual attention and oculomotor control in early infancy
Source: Child Dev. 2024 Oct 24;96(2):619–34. doi: 10.1111/cdev.14185 (PMC11868694; doi:10.1111/cdev.14185)
Supplement: Supplementary file 1 — Data S1. [file CDEV-96-619-s001.docx]

**Supporting Information**

**
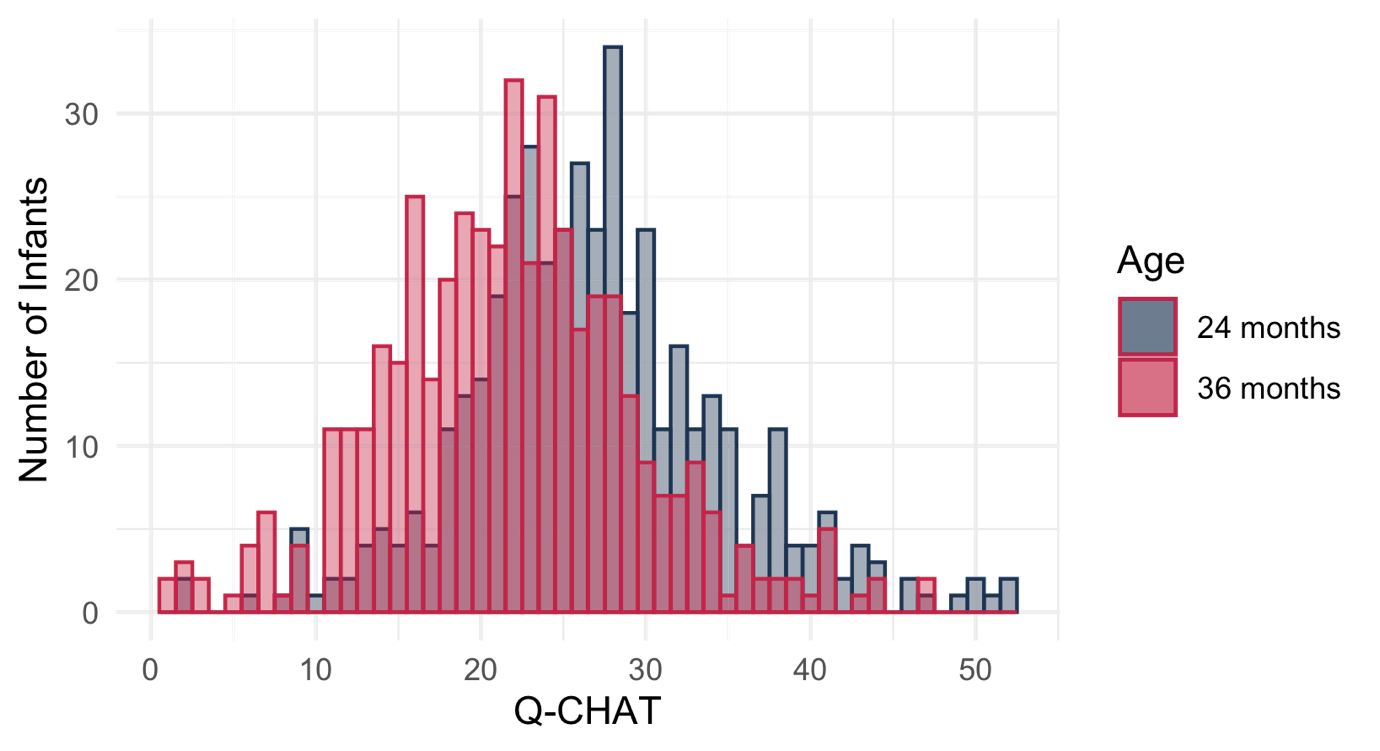
**

**Figure S1.** Comparison of Q-CHAT score distributions for the sample at 24 months (n=308) and at 36 months (n=450). Imputation of missing scores was done to replace missing values (34%) at 36 months (n = 155; imputation was not applied to the 24-month data). This strategy consisted of sequential models based on non-parametric classification and regression tree algorithms (CART; (Gordon et al., 1984)) and was carried out using the mice package in R (Buuren & Groothuis-Oudshoorn, 2011). For further details on the multiple imputation strategy please refer to Bussu & Falck-Ytter (2023).

**Assumption testing for univariate models tested gaze shift latencies from all conditions and difference scores compared to the fully saturated models**

| **Table S1.** Assumption testing for univariate model tested on the gaze shift latency during the gap condition, compared to the fully saturated model. | | | | | | | |
| --- | --- | --- | --- | --- | --- | --- | --- |
| **Model** | **-2LL** | **# parameters** | ***df*** | **AIC** | **Δ LL** | **Δ df** | **p** |
| Fully saturated | 1225.05 | 10 | 440 | 1245.05 | - | - | - |
| Submodel 1 | 1225.28 | 8 | 442 | 1241.28 | .24 | 2 | .89 |
| Submodel 2 | 1231.85 | 7 | 443 | 1245.85 | 6.81 | 3 | .08 |
| Submodel 3 | 1232.79 | 5 | 445 | 1242.79 | 7.74 | 5 | .17 |
| Submodel 4 | 1232.92 | 4 | 446 | 1240.92 | 7.88 | 6 | .25 |
| *Abbreviations: -2LL = log-likelihood fit statistics; df = degrees of freedom; AIC = Akaike Information Criterion;* **Δ** *LL = difference in log-likelihood fit statistics from the reference model;* **Δ** *df = difference in degrees of freedom from the reference model.* | | | | | | | |

| **Table S2.** Assumption testing for univariate model tested on the gaze shift latency during the baseline condition, compared to the fully saturated model. | | | | | | | |  |
| --- | --- | --- | --- | --- | --- | --- | --- | --- |
| **Model** | **-2LL** | **# parameters** | ***df*** | **AIC** | **Δ LL** | **Δ df** | **p** | |
| Fully saturated | 1204.17 | 10 | 440 | 1224.17 | - | - | - | |
| Submodel 1 | 1204.36 | 8 | 442 | 1220.36 | .20 | 2 | .91 | |
| Submodel 2 | 1212.32 | 7 | 443 | 1226.32 | 8.15 | 3 | .04 | |
| Submodel 3 | 1212.64 | 5 | 445 | 1222.64 | 8.47 | 5 | .13 | |
| Submodel 4 | 1212.8 | 4 | 446 | 1220.82 | 8.65 | 6 | .19 | |
| *Abbreviations: -2LL = log-likelihood fit statistics; df = degrees of freedom; AIC = Akaike Information Criterion;*  **Δ** *LL = difference in log-likelihood fit statistics from the reference model;*  **Δ** *df = difference in degrees of freedom from the reference model.* | | | | | | | |  |

| **Table S3.** Assumption testing for univariate model tested on the gaze shift latency during the overlap condition, compared to the fully saturated model. | | | | | | | |
| --- | --- | --- | --- | --- | --- | --- | --- |
| **Model** | **-2LL** | **# parameters** | ***df*** | **AIC** | **Δ LL** | **Δ df** | **p** |
| Fully saturated | 1241.80 | 10 | 440 | 1261.80 | - | - | - |
| Submodel 1 | 1242.97 | 8 | 442 | 1258.97 | 1.16 | 2 | .56 |
| Submodel 2 | 1244.07 | 7 | 443 | 1258.07 | 2.27 | 3 | .52 |
| Submodel 3 | 1245.26 | 5 | 445 | 1255.26 | 3.46 | 5 | .63 |
| Submodel 4 | 1245.80 | 4 | 446 | 1253.80 | 4.00 | 6 | .68 |
| *Abbreviations: -2LL = log-likelihood fit statistics; df = degrees of freedom; AIC = Akaike Information Criterion;*  **Δ** *LL = difference in log-likelihood fit statistics from the reference model;*  **Δ** *df = difference in degrees of freedom from the reference model.* | | | | | | | |
| \| **Table S4.** Assumption testing for univariate model tested on the gaze shift latency during the facilitation condition, compared to the fully saturated model. \| \| \| \| \| \| \| \| \| --- \| --- \| --- \| --- \| --- \| --- \| --- \| --- \| \| **Model** \| **-2LL** \| **# parameters** \| ***df*** \| **AIC** \| **Δ LL** \| **Δ df** \| **p** \| \| \| Fully saturated \| 1244.92 \| 10 \| 440 \| 1264.92 \| - \| - \| - \| \| \| Submodel 1 \| 1245.94 \| 8 \| 442 \| 1261.94 \| 1.02 \| 2 \| .60 \| \| \| Submodel 2 \| 1247.88 \| 7 \| 443 \| 1261.88 \| 2.96 \| 3 \| .40 \| \| \| Submodel 3 \| 1248.97 \| 5 \| 445 \| 1258.97 \| 4.06 \| 5 \| .54 \| \| \| Submodel 4 \| 1248.98 \| 4 \| 446 \| 1256.98 \| 4.06 \| 6 \| .67 \| \| \| *Abbreviations: -2LL = log-likelihood fit statistics; df = degrees of freedom; AIC = Akaike Information Criterion;*  **Δ** *LL = difference in log-likelihood fit statistics from the reference model;* **Δ** *df = difference in degrees of freedom from the reference model.* \| \| \| \| \| \| \| \| | | | | | | | |

| **Table S5.** Assumption testing for univariate model tested on the gaze shift latency during the visual disengagaement condition, compared to the fully saturated model. | | | | | | | |  |
| --- | --- | --- | --- | --- | --- | --- | --- | --- |
| **Model** | **-2LL** | **# parameters** | ***df*** | **AIC** | **Δ LL** | **Δ df** | **p** | |
| Fully saturated | 1239.17 | 10 | 440 | 1259.17 | - | - | - | |
| Submodel 1 | 1241.51 | 8 | 442 | 1257.51 | 2.34 | 2 | .31 | |
| Submodel 2 | 1243.54 | 7 | 443 | 1257.54 | 4.37 | 3 | .22 | |
| Submodel 3 | 1244.14 | 5 | 445 | 1254.14 | 4.97 | 5 | .42 | |
| Submodel 4 | 1247.96 | 4 | 446 | 1255.96 | 8.79 | 6 | .19 | |
| *Abbreviations: -2LL = log-likelihood fit statistics; df = degrees of freedom; AIC = Akaike Information Criterion;*  **Δ** *LL = difference in log-likelihood fit statistics from the reference model;*  **Δ** *df = difference in degrees of freedom from the reference model.* | | | | | | | |  |

**Univariate ACE model fitting on gaze shift latencies from all conditions and difference scores, with sub-models.**

| **Table S6.** Univariate ACE model fitting on the arriving gaze latency during the gap condition, with sub-models. | | | | | | | | | | |
| --- | --- | --- | --- | --- | --- | --- | --- | --- | --- | --- |
| **Model** | **-2LL** | **# parameters** | ***df*** | **AIC** | **Δ LL** | **Δ df** | **p** | **A** | **C** | **E** |
| ACE | 1232.92 | 4 | 446 | 1240.92 | - | - | - | .48 [.06; .61] | .02 [.00; .37] | .50 [.39; .64] |
| **AE** | **1232.94** | **3** | **447** | **1238.94** | **.02** | **1** | **.90** | **.50 [.37; .61]** | **-** | **.50 [.39; .63]** |
| CE | 1238.03 | 3 | 447 | 1244.03 | 5.10 | 1 | .02 | - | .39 [.28; .50] | .61 [.50; .72] |
| E | 1276.04 | 2 | 448 | 1280.04 | 43.12 | 2 | <.001 | - | - | 1 [1; 1] |
| *Abbreviations: -2LL = log-likelihood fit statistics; df = degrees of freedom; AIC = Akaike Information Criterion;* **Δ** *LL = difference in log-likelihood fit statistics from the reference model;* **Δ** *df = difference in degrees of freedom from the reference model; A = % variance explained by additive genetics; C = % variance explained by shared family environment; E = % variance explained by unique environment.* | | | | | | | | | | |

| **Table S7.** Univariate ACE model fitting on the arriving gaze latency during the baseline condition, with sub-models. | | | | | | | | | | |
| --- | --- | --- | --- | --- | --- | --- | --- | --- | --- | --- |
| **Model** | **-2LL** | **# parameters** | ***df*** | **AIC** | **Δ LL** | **Δ df** | **p** | **A** | **C** | **E** |
| ACE | 1213.09 | 4 | 446 | 1221.09 | - | - | - | .60 [.30; .69] | .00 [0; .25] | .40 [.31; .52] |
| **AE** | **1213.09** | **3** | **447** | **1219.09** | **<.001** | **1** | **1.00** | **.60 [.48 .69]** | **-** | **.40 [.31; .52]** |
| CE | 1225.82 | 3 | 447 | 1231.82 | 12.72 | 1 | <.001 | - | .45 [.34; .55] | .55 [.45; .66] |
| E | 1276.04 | 2 | 448 | 1280.04 | 62.95 | 2 | <.001 | - | - | 1[1; 1] |
| *Abbreviations: -2LL = log-likelihood fit statistics; df = degrees of freedom; AIC = Akaike Information Criterion;* **Δ** *LL = difference in log-likelihood fit statistics from the reference model;* **Δ** *df = difference in degrees of freedom from the reference model; A = % variance explained by additive genetics; C = % variance explained by shared family environment; E = % variance explained by unique environment.* | | | | | | | | | | |

| **Table S8.** Univariate ACE model fitting on the arriving gaze latency during the overlap condition, with sub-models. | | | | | | | | | | |
| --- | --- | --- | --- | --- | --- | --- | --- | --- | --- | --- |
| **Model** | **-2LL** | **# parameters** | ***df*** | **AIC** | **Δ LL** | **Δ df** | **p** | **A** | **C** | **E** |
| ACE | 1248.86 | 4 | 446 | 1256.86 | - | - | - | .43 [.20; .56] | .00 [0; .16] | .57 [.44; .72] |
| **AE** | **1248.86** | **3** | **447** | **1254.86** | **<.001** | **1** | **1.00** | **.43 [.28; .56]** | **-** | **.57 [.44; .72]** |
| CE | 1257.95 | 3 | 447 | 1263.95 | 9.09 | 1 | .003 | - | .28 [.15; .39] | .72 [.61; .85] |
| E | 1276.04 | 2 | 448 | 1280.04 | 27.18 | 2 | <.001 | - | - | 1 [1; 1] |
| *Abbreviations: -2LL = log-likelihood fit statistics; df = degrees of freedom; AIC = Akaike Information Criterion;* **Δ** *LL = difference in log-likelihood fit statistics from the reference model;* **Δ** *df = difference in degrees of freedom from the reference model; A = % variance explained by additive genetics; C = % variance explained by shared family environment; E = % variance explained by unique environment.* | | | | | | | | | | |

| **Table S9.** Univariate ACE model fitting on the arriving gaze latency during the facilitation condition, with sub-models. | | | | | | | | | | | |
| --- | --- | --- | --- | --- | --- | --- | --- | --- | --- | --- | --- |
| **Model** | **-2LL** | **# parameters** | ***df*** | **AIC** | **Δ LL** | **Δ df** | **p** | **A** | **C** | **E** |  |
| ACE | 1250.91 | 4 | 446 | 1258.91 | - | - | - | .40 [.14; .53] | .00  [.00; .20] | .60 [.47; .75] |  |
| **AE** | **1250.91** | **3** | **447** | **1256.91** | **<.001** | **1** | **1.00** | **.40 [.25; .53]** | **-** | **.60 [.47; .75]** |  |
| CE | 1257.98 | 3 | 447 | 1263.98 | 7.07 | 1 | .008 | - | .28 [.15; .39] | .72 [.61; .85] |  |
| E | 1276.04 | 2 | 448 | 1280.04 | 25.13 | 2 | <.001 | - | - | 1 [1; 1] |  |
| *Abbreviations: -2LL = log-likelihood fit statistics; df = degrees of freedom; AIC = Akaike Information Criterion;* **Δ** *LL = difference in log-likelihood fit statistics from the reference model;* **Δ** *df = difference in degrees of freedom from the reference model; A = % variance explained by additive genetics; C = % variance explained by shared family environment; E = % variance explained by unique environment.* | | | | | | | | | | | |

| **Table S10.** Univariate ACE model fitting on the arriving gaze latency during the disengagement condition, with sub-models. | | | | | | | | | | | | | | |
| --- | --- | --- | --- | --- | --- | --- | --- | --- | --- | --- | --- | --- | --- | --- |
| **Model** | **-2LL** | | **# parameters** | ***df*** | **AIC** | | **Δ LL** | **Δ df** | | **p** | | **A** | **C** | **E** |
| ACE | 1249.82 | 4 | | 446 | | 1257.82 | - | | - | | - | .44 [.18; .57] | .00 [.00; .18] | .56 [.43; .72] |
| **AE** | **1249.82** | **3** | | **447** | | **1255.82** | **<.001** | | **1** | | **1.00** | **.44 [.28; .57]** | **-** | **.56 [.43; .72]** |
| CE | 1257.96 | 3 | | 447 | | 1263.96 | 8.14 | | 1 | | .004 | - | .28 [.15; .39] | .72 [.61; .85] |
| E | 1276.04 | 2 | | 448 | | 1280.04 | 26.22 | | 2 | | <.001 | - | - | 1 [1; 1] |

*Abbreviations: -2LL = log-likelihood fit statistics; df = degrees of freedom; AIC = Akaike Information Criterion;* **Δ** *LL = difference in log-likelihood fit statistics from the reference model;* **Δ** *df = difference in degrees of freedom from the reference model; A = % variance explained by additive genetics; C = % variance explained by shared family environment; E = % variance explained by unique environment.*

**
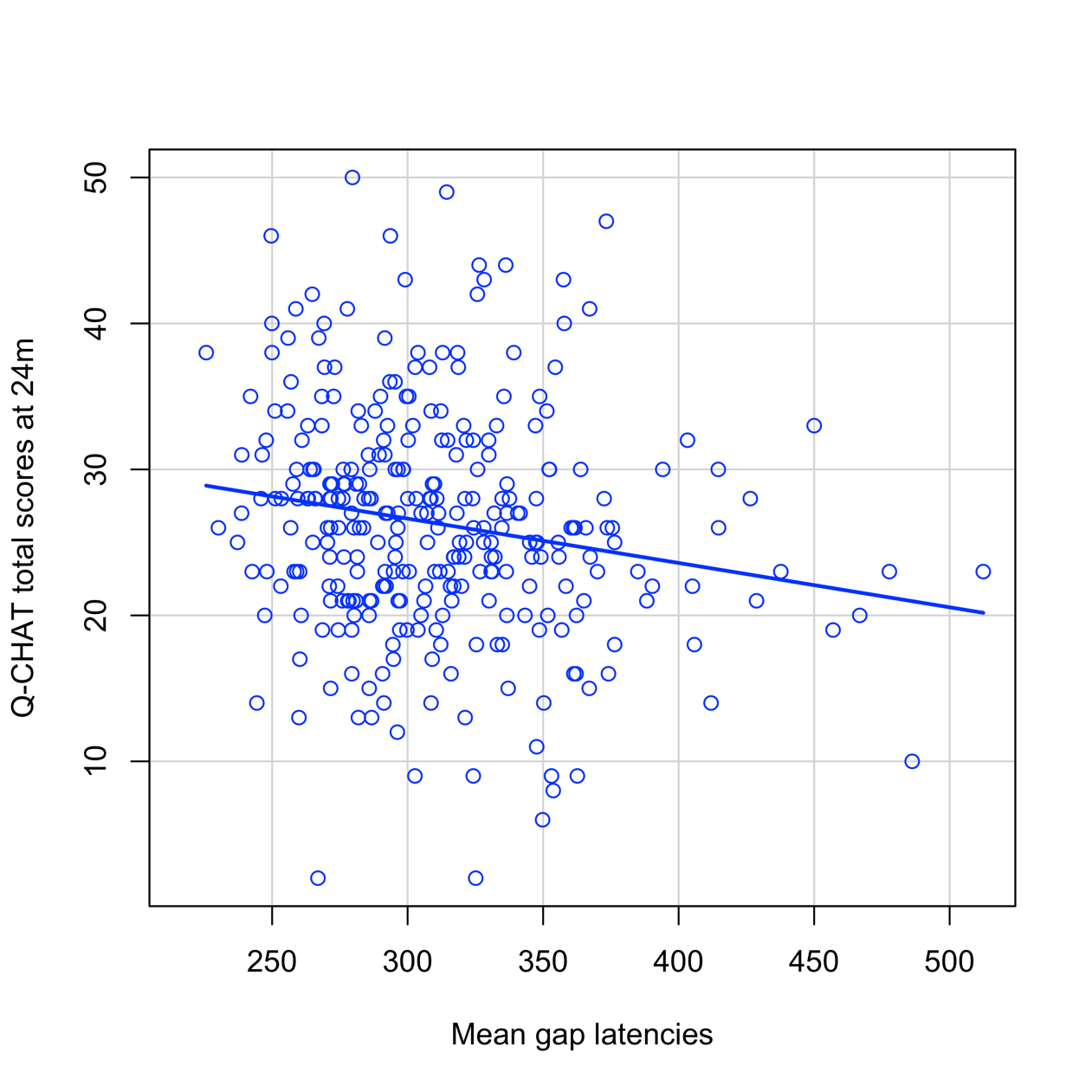
**

**Figure S2.** Association between Q-CHAT scores at 24 months and mean gaze shift latencies (in milliseconds) in the Gap condition (n=308). Please note that this scatterplot is provided for descriptive purposes only; for formal statistical results linked to these data, see the main text.

**Table S11.** Summary statistics of the association analysis between eye-tracking scores and GPS

| **GPS** | **Baseline** | | | **Gap** | | | **Overlap** | | | **Facilitation** | | | **Disengagement** | | |
| --- | --- | --- | --- | --- | --- | --- | --- | --- | --- | --- | --- | --- | --- | --- | --- |
|  | Beta | SE | *p* | Beta | SE | *p* | Beta | SE | *p* | Beta | SE | *p* | Beta | SE | *p* |
| ASD | 6.89 | 4.49 | .13 | 3.38 | 3.19 | .29 | 2.41 | 8.07 | .77 | 3.48 | 2.88 | .23 | -4.45 | 5.9 | .45 |
| ADHD | -1.64 | 4.36 | .71 | -1.68 | 2.79 | .55 | -3.87 | 7.59 | .61 | 0.05 | 3.05 | .99 | -2.24 | 5.62 | .69 |
| SCZ | -1.61 | 4.33 | .71 | 1.1 | 3.04 | .72 | -9.86 | 7.59 | .19 | -2.71 | 3.11 | .38 | -8.26 | 5.82 | .16 |
| IQ | -2.1 | 3.87 | .59 | -1.89 | 2.81 | .5 | -3.76 | 7.19 | .6 | -0.21 | 2.53 | .93 | -1.66 | 5.94 | .78 |
| EA | 0.54 | 4.24 | .90 | 0.47 | 2.98 | .87 | -4.27 | 7.91 | .59 | 0.06 | 3.05 | .98 | -4.8 | 5.9 | .42 |
| Height | 1.74 | 4.65 | .71 | -0.91 | 3.05 | .77 | 0.35 | 7.6 | .96 | 2.66 | 3.28 | .42 | -1.4 | 6.19 | .82 |

The table shows results from the Generalized Estimating Equations (GEE) testing associations between arriving gaze latency scores for the different eye-tracking task conditions tested at 5 months of age and GPS for Autism Spectrum Disorder (ASD), Attention Deficit and Hyperactivity Disorder (ADHD), Schizophrenia (SCZ), Intellectual Quotient (IQ), Educational attainment (EA) and height. Results are reported on separate models in each row and column as regression coefficient (beta), standard error, and uncorrected p-value. Eye tracking variables were residualized by sex, testing age, and socioeconomic status (scaled before being entered into the model). Results are deemed significant for p<alpha with Bonferroni corrected alpha=.002 based on the twenty-five tests performed.

**References**

Bussu, G., & Falck-Ytter, T. (2023). *Missing data strategies in developmental psychology: a practical example on the Babytwins Study Sweden*. https://doi.org/10.31219/osf.io/qzta3

Buuren, S. van, & Groothuis-Oudshoorn, K. (2011). mice: Multivariate Imputation by Chained Equations in R. *Journal of Statistical Software, 45*, 1–67. https://doi.org/10.18637/jss.v045.i03

Gordon, A. D., Breiman, L., Friedman, J. H., Olshen, R. A., & Stone, C. J. (1984). Classification and Regression Trees. *Biometrics, 40*, 874. https://doi.org/10.2307/2530946
